# Post-COVID-19 Syndrome and the Potential Benefits of Exercise

**DOI:** 10.3390/ijerph18105329

**Published:** 2021-05-17

**Authors:** Amaya Jimeno-Almazán, Jesús G. Pallarés, Ángel Buendía-Romero, Alejandro Martínez-Cava, Francisco Franco-López, Bernardino J. Sánchez-Alcaraz Martínez, Enrique Bernal-Morel, Javier Courel-Ibáñez

**Affiliations:** 1 Department of Infectious Diseases, Hospital Universitario Santa Lucía, Cartagena, 30202 Murcia, Spain; amaya.jimeno@carm.es; 2 Human Performance & Sport Sciences Laboratory, University of Murcia, 30720 Murcia, Spain; jgpallares@um.es (J.G.P.); angel.buendiar@um.es (Á.B.-R.); alejandro.mcava@gmail.com (A.M.-C.); francisco.francol@um.es (F.F.-L.); 3 Department of Physical Activity and Sport, University of Murcia, 30720 Murcia, Spain; bjavier.sanchez@um.es; 4 Department of Infectious Diseases, Hospital General Universitario Reina Sofía, University of Murcia, IMIB, 30003 Murcia, Spain; ebm.hgurs@gmail.com; 5 Department of Physical Training, Post-COVID-19 Rehabilitation Unit, Hospital QuirónSalud, 30011 Murcia, Spain

**Keywords:** long COVID, post-COVID-19 syndrome, post-acute sequelae of SARS-CoV-2 infection (PASC), chronic COVID syndrome (CCS), pneumonia, functional capacity

## Abstract

The coronavirus disease (COVID-19), caused by severe acute respiratory syndrome-coronavirus-2 (SARS-CoV-2) infection, is leading to unknown and unusual health conditions that are challenging to manage. Post-COVID-19 syndrome is one of those challenges, having become increasingly common as the pandemic evolves. The latest estimates suggest that 10 to 20% of the SARS-CoV-2 patients who undergo an acute symptomatic phase are experiencing effects of the disease beyond 12 weeks after diagnosis. Although research is beginning to examine this new condition, there are still serious concerns about the diagnostic identification, which limits the best therapeutic approach. Exercise programs and physical activity levels are well-known modulators of the clinical manifestations and prognosis in many chronic diseases. This narrative review summarizes the up-to-date evidence on post-COVID-19 syndrome to contribute to a better knowledge of the disease and explains how regular exercise may improve many of these symptoms and could reduce the long-term effects of COVID-19.

## 1. Introduction

By 10 April 2021, over 127 million cases of Coronavirus disease (COVID-19) infection have been reported worldwide [1]. The pandemic is caused by the severe acute respiratory syndrome coronavirus 2 (SARS-CoV-2), a betacoronavirus responsible of a new type of acute respiratory infection and atypical pneumonia with the potential to evolve into a severe acute respiratory syndrome (SARS), which was first described in Wuhan province, China, on 10 December 2019. Between 10 and 20% of COVID-19 patients with symptomatic acute COVID-19 will evolve to a persistence phase of clinical manifestations lasting more than one month [2], with chronic ailments such as fatigue, post-exertional malaise, dyspnoea, headache, and many other neurocognitive conditions described as brain fog, inability to perform daily physical tasks [3] and increased likelihood of developing stress, depression, irritability, insomnia, confusion or frustration [4]. This condition, defined as post-COVID-19 syndrome, is increasingly affecting a high number of people as the pandemic evolves. 

Tremendous efforts have been made in the study of SARS-CoV-2 infection and protection, despite leading to evidence-based mitigation strategies [5] and next-generation vaccines [6]. Today, many pharmacologic therapies have been used, but few have shown the true impact on survival, and none, to date, have been shown to reduce the sequelae of the disease or specifically the evolution to persistent symptoms. Unfortunately, the COVID-19 pandemic consequences will not be fully solved even with zero cases and global vaccination achieved. In particular, the effective long-term management of the effects of post-COVID-19 syndrome is a challenge that requires awareness.

Overwhelming evidence exists that exercise produces short-, middle- and long-term health benefits that prevent, delay, mitigate and even reverse a large number of metabolic, pulmonary, cardiovascular, neurocognitive, inflammatory, rheumatic and musculoskeletal diseases [7,8,9,10,11,12,13]. Accordingly, physical inactivity has been associated with a higher risk for severe COVID-19 outcomes [14]. Likewise, high levels of cardiorespiratory fitness are shown to reduce the likelihood of hospitalization due to COVID-19 [15]. Therefore, it could be hypothesized that an optimal exercise prescription would benefit individuals with persistent COVID-19 symptoms.

Despite there being no data about the benefits of exercise in post-COVID-19 syndrome, the latest recommendations emphasize the need for symptom-titrated physical activity and tailored exercise in rehabilitation [16]. Thus, proper and tailored exercise stands as a promising, effective therapy for mitigating the post-COVID-19 symptoms and helping people in recovering faster and increasing their autonomy, functionality and quality of life [17]. 

This narrative review provides a summary of the latest available information on post-COVID-19 syndrome and describes how physical exercise could provide symptomatic relief for many of the patients with this condition. 

### 1.1. Definition of the Post-COVID-19 Syndrome 

**Figure 1:**
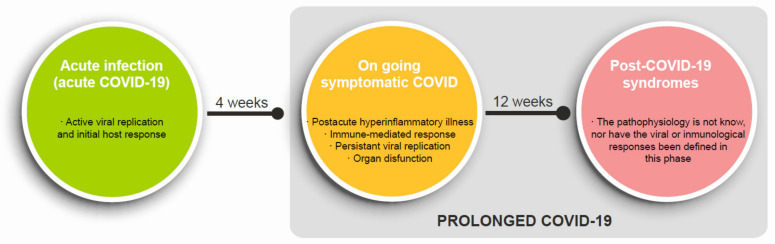
Physiopathology characterization of the different evolutionary phases of SARS-CoV-2 infection to post-COVID-19 syndrome.

The UK National Health Service (NHS) defined the post-COVID-19 syndrome as unexplained, persisting signs or symptoms over 12 weeks, developed during or after the COVID-19 infection [18] Prolonged COVID-19 is commonly used to describe signs and symptoms that continue or develop after acute COVID-19. It includes continuous symptomatic COVID-19, called ongoing symptomatic COVID-19 (4 to 12 weeks), and post-COVID-19 syndrome (≥12 weeks) (Figure 1). The true prevalence of post-COVID-19 syndrome is not known yet. According to data from the UK’s Office for National Statistics (ONS) on 1 April 2021, of more than 20,000 people who tested positive between 26 April 2020 and 6 March 2021, mainly non-hospitalized (90%), 13.7% persisted symptomatic after 12 weeks of evolution [19].

The term long COVID has been used since May or June 2020 [20] to define those patients who present symptoms once the acute phase of the disease is over. Other denominations are common, such as chronic COVID-19 syndrome (CCS), post-COVID-19 syndrome or post-acute sequelae of SARS-CoV-2 infection (PASC). Another more popular name such as long hauler has been used, thus describing the long duration of the symptoms occurring in these patients. 

The current 12-week window is based on a median duration of symptoms disappearance. Even when prolonged viral ribonucleic acid (RNA) detection has been shown for months, the median duration of viral detection in respiratory samples is 18 days [21], and estimates of four weeks to define the acute phase of the infection look reasonable. Additionally, the possibility of recovering viable and cultivable virus beyond the 10th day after the infection begins, in mild patients whose symptoms have disappeared, is an exception [22]. It is important to note that these definitions do not imply that the disease has ended or that people have recovered, but that the acute phase of the disease has finished. 

Other authors [23,24] have developed a theoretical framework in which the patient with SARS-CoV-2 infection could go through different stages during the evolutionary course of the disease. They attribute physiopathological and/or laboratory diagnostic characteristics to each to define them. Thus, a patient could experience all or several of the stages over time (Figure 1): after acute COVID-19, one may evolve to residual symptoms that persist after recovery (convalescence or ongoing COVID-19), to organ dysfunction that persists after the acute phase, including PICS (post-intensive care syndrome) and other sequelae, and to develop new symptoms or syndromes (truly post-COVID-19 syndrome).

However, other differential diagnoses could hinder the diagnosis of post-COVID-19 syndrome, for instance, with atypical presentations in patients with comorbidities, adverse events of the drugs used, other post-infectious syndromes (over-imposed healthcare-related infection or a coexisting bacterial or viral co-infection) and even psychological alterations related to the course of COVID-19 [25]. Likewise, the possibility of reinfection (an infection caused by a strain genetically confirmed as different from the first and separated by at least three months from the first event) could simulate persistent COVID-19 [26] or even symptoms, of variable duration, of reactogenity following receipt of mRNA-based COVID-19 vaccines, which can stimulate systemic reactions including fatigue, headache, myalgia or chills [27]. 

Therefore, the key to diagnosis is probably not so much to establish a specific timeframe but rather a continuum of symptoms, which could be variable depending on the individual, as long as there is clinical certainty of the absence of another intercurrent process that could explain them. After acute COVID-19, one can evolve to residual symptoms that persist since then as a result of organ failure established beyond the acute phase or to the appearance of new symptoms or syndromes that develop after an initial infection independent of the severity of the presentation, even if it was asymptomatic or mild. These two categories are not mutually exclusive, with the latter representing the real post-COVID-19 syndrome.

### 1.2. Clinical Manifestations of Post-COVID-19 Syndrome

Soon after the beginning of the pandemic, it was estimated that symptoms disappeared within approximately two weeks in mild cases and between 3 and 12 weeks in more serious ones [28]. It has subsequently been seen that these periods of time are indicative and depend largely on age, form of presentation and pre-existing comorbidities [29].

Case reports and cross-sectional studies (i.e., online survey) report a list of more than 200 different symptoms in the evolution of post-COVID-19 syndrome [30,31]. Considering the multiorgan nature of the syndrome, it should be pointed out that it can produce almost any clinical manifestation. 

**Figure 2:**
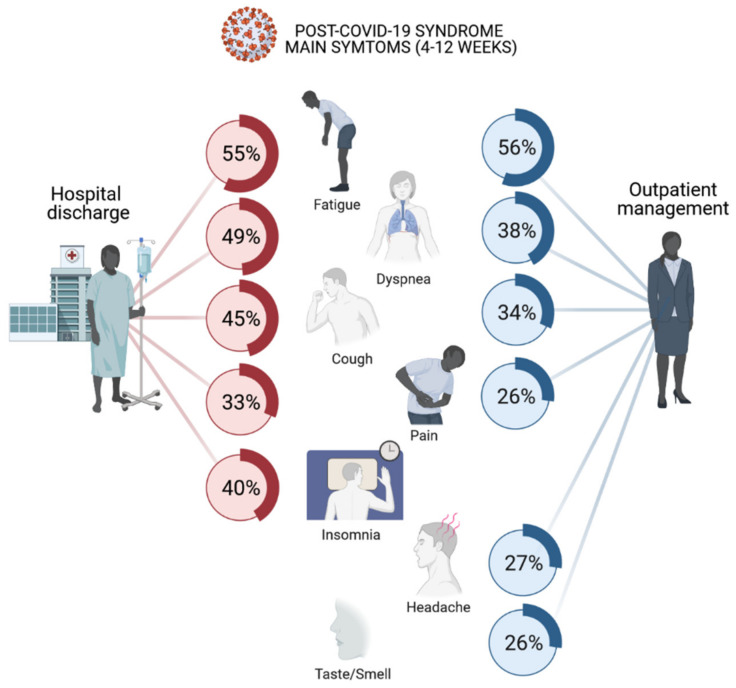
Prevalence of main symptoms in post-COVID-19 syndrome, hospitalized [40,41,42,43,44,45,46,47,48,49,50] or outpatients [51,52,53,54,55,56], evaluated between 4 and 12 weeks after diagnosis. The mean of the presentation of symptoms appears considering all the studies that contribute.

To date, three meta-analyses and a systematic review on the long-term effects of COVID-19 have been released, all on non-peer-reviewed pre-prints [32,33,34]. In Figure 2, we summarize the frequency of the presentation of symptoms, depending on whether or not the patients required hospitalization. Breathlessness, persistence of smell and taste disturbances, fatigue and neuropsychological symptoms (headache, memory loss, slow thinking, anxiety, depression and sleep disorders) were the most frequently reported. Musculoskeletal symptoms were also frequent. It should be noted that the quality of the studies from which the information is extracted is low, and so is the power of the conclusions (mostly cohort studies, in some of which, the main objective was not the nature of symptoms, and in which the method of recruitment was variable, including self-selection surveys (high risk of bias)). There are few prospective studies with assessments beyond 12 weeks [31,35,36,37,38,39] fulfilling the NHS current criteria for post-COVID-19 syndrome.

Other characteristics that might be identified with post-COVID-19 syndrome are:

- Symptoms are commonly associated: 80% of patients have more than one [32], usually more than two and up to more than 10 [30,31,49,52,57].
- Women tend to be more affected than men [30,31,50,51,52]. The association between fatigue and myalgias is significantly more frequent in females than males. This phenomena has not been found in other symptoms cluster including low mood, anxiety, and sleep disturbance or in a cluster consisting of comprised memory impairment, attention deficit, and cognitive impairment [38].
- Persistent symptoms are more likely to appear with increasing age [31,52,53,58] and with severity of presentation [29,52,58]. After discharge, fatigue, dyspnoea and neuropsychological perturbances were significantly more frequent in ICU patients. Not all studies agree on a relationship with previous comorbidities.
- The symptoms are of a fluctuating nature and their appearance varies over time [59]. Patients’ perceptions were generally changeable both in time and form (relapsing-remitting). There is not a defined evolution pattern of the symptoms and patients usually respond to different stimuli (mainly physical or mental activity and stress).
- Overall, patients improve spontaneously over time [31,52,58,59] although continuous patterns in time and intensity have been described and some patients worsen with the course of the disease [31].
- Recently, some studies has suggested that it is possible to identify the immunologic signatures of post-COVID-19 syndrome [60].
- Fatigue is, along with neurocognitive symptoms, the most debilitating feature and has the greatest impact on post-COVID-19 syndrome patients’ quality of life [30,31,50,51]. Patients describe these symptoms as limiting, seriously affecting the development of normal working activity [31,50,61]
- It is noteworthy that in several studies, both patients with confirmatory diagnostic tests, and those who could not be tested but their case was suspected, or even those who tested negative, have no significant differences in the presentation or the evolution of symptoms [30,31,59].
- Currently, there is no evidence supporting the notion that prolonged COVID-19 is equated to myalgic encephalomyelitis and/or chronic fatigue syndrome (ME/CFS). Both prolonged COVID-19, ME and CFS are heterogeneous and difficult to characterize, and while some patients with prolonged COVID-19 syndrome may meet diagnostic criteria for EM/CFS, there is a significant population with persistent fatigue that does not meet these criteria; therefore, further research is needed. Identification between the two syndromes could constitute a risk of avoiding other pathologies, complications or sequelae or erring in the management of the syndrome.

## 2. Physiopathology of the Post-COVID-19 Syndrome 

Emerging data suggest that multiple and/or synergistic causal mechanisms may underlie post-COVID-19 syndrome. Genetic host factors, pre-existing lesions of COVID-19 targeting organs due to comorbidities and acute COVID-19 organ failure itself, may be involved. Modifications in the immune response to the virus and viral mechanisms can be added, such as the establishment of reservoirs, latency states or even the potential viral integration in the host genome [62]. In addition, unknown or poorly defined factors may influence the appearance of prolonged symptoms, as biopsychosocial circumstances related with COVID-19. 

### 2.1. Immune System 

Evidence suggests that severe forms of presentation are characterized by innate pathological immune activation and an exaggerated and poorly directed host immune response [63], sometimes as a cytokine storm, which causes multiorgan failure and thus may cause persistence of symptoms. It would also be possible that self-limiting forms may differ from persistent forms in their pattern of immune activation or on their autoantibody generation [64]. The dysregulation of the immune response also affects the physiological functions of the vascular endothelium (endothelitis) and underlies many of the complications in COVID-19, mainly of thromboembolic nature [65]. Accordingly, the persistence of endothelial inflammatory mechanisms might mediate the appearance of chronic symptoms.

### 2.2. Cardiopulmonary Sequelae

An altered diffusion capacity of the lungs for carbon monoxide (DLCO) and restrictive patterns are the more common functional impairments found in COVID-19 pneumonia survivors in the long term [66]. A prospective, longitudinal and observational study performed in symptomatic after recovery from acute SARS-CoV-2 individuals were evaluated by MRI more than three months after diagnosis (only 18% had required hospitalization); two thirds of them (66%) had persistent lesions of different entity in one or more organs, mainly in the heart and lungs [57]. The researchers also assessed symptoms by standardized questionnaires and organ function (heart, lungs, kidneys, liver, pancreas, spleen). Fatigue, muscle aches, breathlessness and headaches were frequently reported by these patients, as in post-COVID-19 syndrome. These findings suggest the possibility of residual organ involvement, even in mild scenarios, as a cause of the long-term symptoms, at least in a non-negligible percentage of patients. 

### 2.3. Neurological and Biopsychosocial Disturbances 

It is well-established that survivors of COVID-19 can develop anxiety, depression, sleep disturbances and post-traumatic stress disorder (PSTD) [42,50,51,67]. PSTD may be responsible for severe distress or clinically significant impairment in the social, occupational and other basic areas of functioning of the individual. In addition, it is often associated with the appearance of physical symptoms such as fatigue and/or dyspnea, palpitations, chest pain, headache, sweating, nausea, tremors, insomnia and various neuropsychological disorders such as mental dullness or emotional flattening, which may contribute to the misinterpretation of the prolonged symptoms of COVID-19. 

It is now clear that quarantine has had a very perdurable effect on psychological and social health and that the weight of these mood alterations (loneliness, fear, anxiety, depression, acute stress and insomnia) falls especially among the most vulnerable populations: those with pre-existing mental and chronic health disorders, health care workers and those living in the most restricted areas and those who are poor [68]. The neuropsychiatric symptomatology of post-COVID-19 syndrome focuses precisely on the loss of these cognitive abilities. These effects that patients described as brain fog, memory perturbances and sleep manifestations [31] are consistent with poor attention or concentration, difficulty thinking, difficulty with executive functioning (planning, organizing, figuring out the sequence of actions, abstracting), slowed thoughts, short- and long-term memory impairments, speech and language issues and difficulty with sleep (insomnia, night sweats, restless legs, etc.). Neurocognitive impairment could be related to the neurotropic properties of this virus, allowing it to infect brain regions. Different mechanisms are proposed to this, such as neuronal retrograde invasion through olfactory bulb and/or trigemic nerve, through blood (exceeding the blood–brain barrier (BBB)) and through immune-mediated pathways (immune cells that transmit the pathogen into the brain). Other indirect causes of brain lesion could be through a local or systemic abnormal inflammatory response causing endothelial damage, increased permeability of the BBB, or access of pro-inflammatory cytokines, which would damage brain homeostasis and finally induce neuronal death [69]. Other neurological manifestations of long COVID-19 are secondary to disruption of the autonomic nervous system (dysautonomia), resulting in orthostatic intolerance syndrome, also known as postural orthostatic tachycardia syndrome (POTS), provoking palpitations, irritable bowel or recurrent presyncopal episodes [70]. 

## 3. Treatment of the Post-COVID-19 Syndrome

To date, there is still no specific treatment for the management of patients with post-COVID-19 syndrome. The greatest research effort has rightly focused on prevention and treatment of the acute phase of the disease. Future research, focusing on medical as well as social aspects, must consider the disease continuum, including prolonged forms.

At the time of this review, less than fifteen clinical trials had been registered with the aim of addressing the treatment of post-COVID-19 syndrome or long COVID-19: six on drugs (montelukast (NCT04695704), naltrexone plus NAD+ (NCT04604704), leronlimab (NCT04678830), ruconest (NCT04705831), LYT-100), dietary supplements (dietary Supplement: ADAPT-232 oral solution (NCT04795557) and Niacine (NCT04809974), one on hyperbaric oxygen (NCT04647656) and one through a supervised exercise intervention (NCT04718506), out of a total of 5273 studies for COVID-19 research [71]. None of them have been completed so far. 

Although the evolution will be spontaneous to resolution in most of cases, because of how exasperating this condition can be, it requires a continuity of care through a multidisciplinary and holistic approach. Some published guides and recommendation documents, with a complete vision of the patient, have addressed aspects of a physical, psychological and social nature [2,18]. Once the presence of systemic complications (persistent organic damage or PICS) and the need for referral to certain specialists has been ruled out, care should focus on symptomatic management and physical and mental rehabilitation (including fatigue management, respiratory retraining or psychological or psychiatric support) [18]. The perceptions and autonomy of the patient must be at the centre of shared decision-making regarding the scheduling of care for these patients and their health status. 

Comprehensive approaches have not been considered despite the fact that COVID-19 is a clearly multisystemic disease with a non-linear evolution and long-term implications. No study, to date, has addressed treatment schemes for these patients, possibly because the underlying pathophysiological is not known or even if it is the same in all patients. Besides, among the problems of not having a clear diagnostic identification is the impossibility of planning the best therapeutic approach, but also the origin of problems of a more personal nature such as not being able to access the resources for the management of COVID-19 (most patients with persistent symptoms will be mild patients who did not require hospitalization) or as belonging to a limbo of patients with complex syndromes, such as CFS, which in general, have little diagnostic and research interest. 

There is no current evidence regarding the role of a nutritional approach or physical exercise in the symptomatic recovery of these patients. As a multidisciplinary team dedicated to the study of the effects of physical exercise on health, we consider that this is an essential tool in the management not only of acute disease, but especially of long-term manifestations. 

## 4. The Potential Role of Exercise in Post-COVID-19 Syndrome

The COVID-19 syndemic (a situation generated by the convergence of an infectious disease, the presence of other chronic non-communicable diseases, such as obesity, and the existence of social determinants, which affect the health of the population) has exposed society to a stressful situation never seen before, at a time in which it already had a highly compromised disposition when it comes to population levels of physical activity and exercise. According to the Centers for Disease Control and Prevention (CDC), less than a quarter of the American adult population of both sexes reported performing a sufficient amount of both resistance and strength exercise to fulfil the basic indications referred in the health guidelines [72].

The confinement, the subsequent perimeter closures of the cities and the limitation of urban mobility along with the cessation of all types of group activities, the interruption of non-professional team sports and many other recreational options related to movement, such as parks and leisure areas or swimming pools, have further deteriorated the condition of citizens. After the confinement, there has been a supposed return to normality, in which, on many occasions, previous activities have not been recovered, especially in people who has suffered COVID-19.

**Figure 3:**
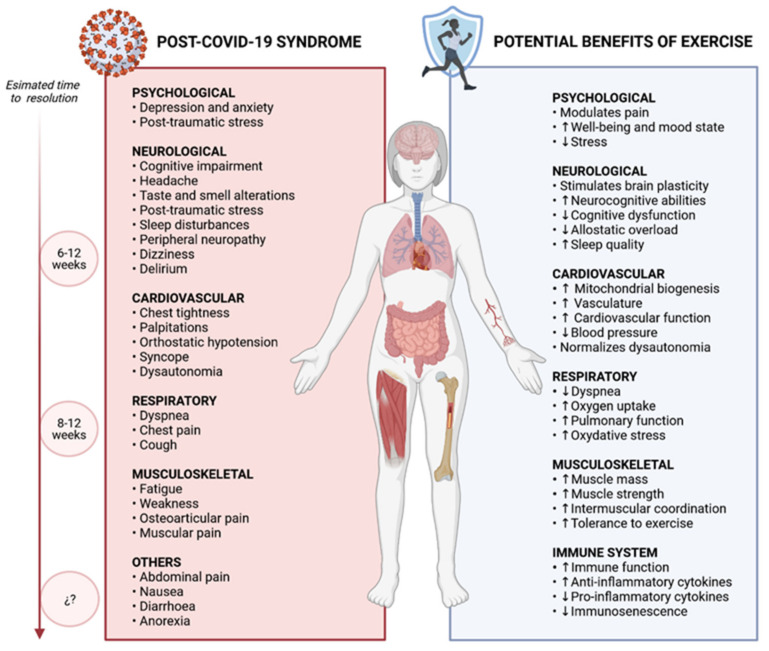
Potential benefits of exercise on the most frequent clinical manifestations of post-COVID-19 syndrome.

Therefore, it is necessary not only to recover physical exercise in the inactive population, but also to position it as a tool in the management of patients with post-COVID-19 syndrome. Given that exercise has been shown to be beneficial in multiple pathologies with which the post-COVID-19 syndrome shares similarities both in terms of symptoms and its possible pathogenic mechanisms, it is worth considering the potential favourable effect that this would bring in the recovery of these patients. Figure 3 illustrates the potential benefits of exercise on the most frequent clinical manifestations of post-COVID-19 syndrome.

### 4.1. Exercise Is Beneficial for Immunological Health

Contrary to traditional beliefs, exercise is not detrimental to immune competency but rather can act as an adjuvant to stimulate the immune system by inducing mitochondrial adaptations, cell generation and immune surveillance [73,74,75,76]. Physical fitness status can be a determining modifiable factor for the promotion of metabolic and functional adaptations in T lymphocytes and monocytes, counteracting inflammatory environments caused by expanded adipose tissue and sedentary behaviour, as well as delaying the immunosenescence caused by aging [77]. Regular release of muscle-derived anti-inflammatory cytokines (IL-6, IL-7, IL-10, IL-15), linked with the inhibition of pro-inflammatory cytokines (IL-1β, IL-18, TNF-α), have been purported to play important roles in the beneficial effects of exercise on immunity [75,78,79]. Indeed, there is growing evidence suggesting an anti-carcinogenic effect of exercise through direct changes in circulating proteins, RNA molecules and metabolites [80].

Exercise attenuates immunosenescence by maintaining the peripheral T-cell pool and natural killer cell compartments, and it seems likely to improve the immune response to SARS-CoV-2 antigens [81]. In support of this argument, it has been recently demonstrated that regular, moderate to vigorous physical activity reduced the risk of community-acquired infectious diseases and infectious disease mortality [82]. All in all, it should be noted that exhaustive and excessive exercise training may cause mitochondrial functional impairment, inducing a dysregulated systemic inflammatory response, thus being detrimental for health [83,84,85]. Hence, individually tailored exercise prescriptions among people with post-COVID-19 are essential to elicit positive adaptive changes to enhance immune function. 

### 4.2. Exercise Helps to Manage and Mitigate Physical Syndromes

Individual and targeted exercise is highly recommended as a non-pharmacologic strategy for treating rheumatic and musculoskeletal diseases, characterized by chronic pain, muscle weakness, physical limitations, fatigue and low tolerance to exercise [11,86,87,88,89]. Besides, strength training and multicomponent exercise programs have been extensively demonstrated as safe and effective among vulnerable people in reversing frailty and weakness and restoring functional capacity in the short- and long-term [12,90,91,92,93]. In particular, exercise has been demonstrated to confer protection against functional deterioration in institutionalized older adults during COVID-19 lockdown/confinement situations [93]. Indeed, strength training confers unique, multi-systemic benefits to the musculoskeletal system, improving both morphological (increasing number of sarcomeres in parallel, increasing the synthesis of contractile assemblies of actin and myosin and altering muscle fibres’ composition) and neural (improving neurological system and intermuscular coordination) factors and regulating the whole body metabolism [94]. 

Of interest, the latest evidence on strength training support that using low loads, low volume and not-to-failure repetitions produce considerable improvements in maximal dynamic strength, power output and muscle hypertrophy while preventing typical discomfort, fatigue or stiffness after traditional high-demanding training [95,96,97]. This is important given that people with post-COVID-19 syndrome are expected to be reluctant to engage in exercise due to fatigue, deconditioning and low tolerance to exercise intensity. Thus, motivating patients to start with an exercise programme is a key challenge. However, whereas entering an exercise program is physically and mentally challenging in people with chronic pain, once the practice starts, making exercise a pleasant, fun and well-tolerated experience contributes in setting the routine and eventually achieving health improvements [98,99,100]. Accordingly, maintaining the motivation and monitoring intensity is of the utmost importance among people with post-COVID-19 syndrome to avoid overtraining by working out within individuals limits and allowing enough recovery time between efforts.

### 4.3. Exercise Is an Effective Treatment for Pulmonary Complications 

Pulmonary rehabilitation relieves dyspnea and fatigue, and enhances self-autonomy of people with pulmonary disorders such as interstitial lung disease [101] and chronic obstructive pulmonary disease (COPD) [102]. Indeed, initiating a pulmonary rehabilitation is associated with lower risk of mortality among people hospitalized for COPD [103]. Both continuous and interval endurance training improve cardiopulmonary outcomes; however, interval-based training is used as a more effective approach to optimise the load that can be tolerated in exercise programs for clinical pulmonary patients [104]. In addition, muscle dysfunction as a consequence of lung disease (muscle atrophy, reduced oxidative capacity and a decreased proportion of type I muscle fibres) can be mitigated and even reversed with strength training interventions [105,106].

Accordingly, concurrent training (endurance and strength) stands as the preferable treatment for lung diseases to improve peak pulmonary oxygen uptake, systematic oxidative stress, muscle strength, muscle size, functional capacity, and quality of life [107,108]. It has been recently described that a pulmonary rehabilitation program after hospitalization improved respiratory function, quality of life, mobility and psychological function in older adults with COVID-19 [109]. Despite its benefits being beyond any doubt, the current worldwide availability of pulmonary rehabilitation services are alarmingly low [110], which may have negative implications in the post-COVID-19 management. 

### 4.4. Exercise Improves Cardiovascular Health 

There is plenty evidence that exercise is an essential therapeutic tool to improve cardiovascular health through enhancing mitochondrial biogenesis and function, restoring and improving vasculature (cardiac remodelling, angiogenesis, blood volume expansion), and the release of myokines from skeletal muscle that preserve or augment cardiovascular function [111]. It is well-known that structured rehabilitation programs after cardiac disease lead to improvements in mortality, hospital readmission, cardiopulmonary fitness and functional status [112,113,114]. Likewise, regular, supervised aerobic exercise training is known to be an excellent nonpharmacological treatment of hypertension [115,116]. 

The benefits of these physical and educational programs in patients with chronic cardiopulmonary disorders have also been shown from the psychosocial point of view, improving quality of life and mood. Even more, moderate targeted exercise should be prescribed in all individuals with cardiovascular disease [117].

### 4.5. Exercise Stimulates Brain Plasticity and Increases Psychological Well-Being 

Exercise can act as a psychoactive drug [118]. Regular exercise has been proven to be a powerful tool in improving the quality of life, in controlling mood and its illnesses (depression and anxiety), in reducing psychological stress and in modulating the perception of pain [119]. Exercise modulates the brain structure and function to stimulate a healthier neurological phenotype [120]. The evidence is uncountable when evaluating the beneficial effects of physical exercise in the evolution of neurodegenerative diseases (for example in Alzheimer’s disease [121]) because it has a role in improving different neurocognitive abilities such as memory and learning, concentration, inhibitory control, cognitive flexibility and processing information. 

Exercise induces neurochemical and structural changes mainly through contraction-induced myokines release and brain-derived neurotrophic factor (BDNF), provoking neurogenesis and synaptogenesis, particularly in the dentate gyrus of the hippocampus, which promotes brain plasticity and prevent cognitive dysfunction [120]. Indeed, exercise has proven its effectiveness in dysautonomia and POTS, quite frequent among people with post-COVID-19 syndrome, by restoring upright hemodynamics, normalizing renal-adrenal responsiveness, and improving quality of life [122,123]. In addition, recent cross-section studies support the need of regular moderate exercise as a resilience factor to reduce COVID-related allostatic overload by improving mental and physical well-being [124].

### 4.6. The role of Advanced Training Methods for Health 

Advancements on exercise sciences’ skills, knowledge and technology has grown immensely over the last years. New trends in resistance and cardiopulmonary training methods appeal to more individualized regimes with a variety of exercises, stimuli and intensities, which are controlled by incorporating new technologies and monitoring tools. Current training methods are based on new approaches to monitoring exercise intensity (e.g., rate of force development, movement velocity), technical execution (e.g., biomechanics, electromyography), readiness (e.g., heart rate variability) and physiological responses (e.g., ventilation, lactate, core temperature, heart rate, muscle oxygen saturation) while providing biofeedback in real time, ensuring the accomplishment of targeted results [125,126,127,128,129,130]. Likewise, a number of portable devices, wearable tools and apps for physical activity and fitness are emerging, making remote control of the patients’ evolution possible [131]. In addition to aiding professionals in providing targeted exercise programmes, these developments may represent an added value for the pandemic to carry out high-quality, supervised home-based trainings during particular restrictive situations (such as lockdown periods) or people with severe mobility limitations. Due to the multiorgan nature of the post-COVID-19 syndrome, there is a need to conduct systematic and multifactorial evaluation of the physical and functional capacity to facilitate the prescription of tailored exercise programmes. To this purpose, a cross-disciplinary approach including exercise sciences know-how and experience has been proposed as essential to effectively response to this health crisis [17]. 

## 5. Concluding Remarks

There is sufficient evidence suggesting that tailored and supervised exercise training may be an effective multisystemic therapy for post-COVID-19 syndrome that suits the diversity of the cases and symptoms. Further examination on the effects of exercise-based treatments on post-COVID-19 syndrome are required to give practical insights about what type of exercise should be preferably prescribed, with emphasis on intensity and load management and adherence strategies. Furthermore, the impact of post-COVID-19 syndrome on certain special population groups such as children, adolescents or institutionalized older adults remains unknown.

A multidisciplinary and integrative approach including exercise sciences is essential, where clinical conditions are addressed but must integrate neurocognitive and psychological aspects into the assessment, as well as the social impact that this pathology entails. New proposals for research in the prolonged phase of the disease and all efforts that enable a complete functional recovery and a return to a previous life are warranted.

## Data Availability

Not applicable.
